# Complete Circular Genome Sequences of Three Bacillus cereus Group Strains Isolated from Positive Blood Cultures from Preterm and Immunocompromised Infants Hospitalized in France

**DOI:** 10.1128/MRA.00597-21

**Published:** 2021-10-14

**Authors:** Mariem Ben Khedher, Fredrik Nindo, Alicia Chevalier, Stéphane Bonacorsi, Gregory Dubourg, Florence Fenollar, Florence Casagrande, Romain Lotte, Laurent Boyer, Armel Gallet, Jean-Marc Rolain, Olivier Croce, Raymond Ruimy

**Affiliations:** a Bacteriology Laboratory, Archet 2 Hospital, CHU, University Côte d'Azur, Nice, France; b Institute for Research on Cancer and Aging of Nice (IRCAN), CNRS UMR 284, INSERM U1081, Université Côte d'Azur, Nice, France; c Aix Marseille University, IRD, AP-HM, MEPHI and IHU-Méditerranée Infection, Marseille, France; d Université Côte d'Azur, INSERM U1065, C3M, Nice, France; e IAME, UMR 1137, INSERM, Université de Paris, AP-HP, Paris, France; f Laboratoire de Microbiologie, Hôpital Robert Debré, AP-HP, Paris, France; g Department of Neonatal Reanimation, University Côte D'Azur, CHU de Nice, Nice, France; h Université Côte d'Azur, CNRS, INRAE, ISA, UMR CNRS 7254/INRAE 1355/UCA, Sophia Antipolis, Nice, France; University of Maryland School of Medicine

## Abstract

We report here the complete genome sequences of three Bacillus cereus group strains isolated from blood cultures from premature and immunocompromised infants hospitalized in intensive care units in three French hospitals. These complete genome sequences were obtained from a combination of Illumina HiSeq X Ten short reads and Oxford Nanopore MinION long reads.

## ANNOUNCEMENT

The Bacillus cereus group comprises 18 closely related valid species, including B. cereus sensu stricto, that can cause extradigestive human infections, which are particularly severe in immunocompromised or premature infants (1–3). Since 2019, the literature on bacteremia in premature neonates has reported an increase in B. cereus infections. Matrix-assisted laser desorption ionization–time of flight mass spectrometry (MALDI-TOF) is widely used for bacterial identification, although its power of discrimination between closely related species is insufficient (4). Genome sequencing appears to be the best solution for proper identification. From 2013 to 2017, three strains identified as B. cereus based on MALDI-TOF were isolated from positive blood cultures from one immunocompromised infant and two premature infants hospitalized in intensive care units in three French hospitals. Colonies were obtained on 5% blood agar plates (Becton, Dickinson, Heidelberg, Germany) after incubation under an aerobic atmosphere at 35 ± 2°C for 24 h. Genomic DNA (gDNA) was extracted from pure colonies of the strains using a QIAamp DNA kit (Qiagen) according to the manufacturer’s instructions, with the addition of 100 μg/ml of lysozyme. Whole-genome sequencing (WGS) libraries were prepared according to the manufacturer’s protocols for Illumina libraries. Briefly, 1 μg genomic DNA was randomly fragmented and purified. The fragments were then end repaired, followed by A-tailing and ligation of the Illumina adaptors. After size selection, several rounds of PCR amplification were performed to enrich the adapter-ligated DNA fragments. After purification, the qualified libraries were sequenced in 150-bp paired-end format using the HiSeq X Ten platform. For Nanopore sequencing, the gDNA was not sheared, and size selection was performed using a BluePippin instrument (Sage Scientific) of 10 to 50 kb. The Nanopore libraries were generated according to the manufacturer’s instructions (genome DNA by ligation sequencing kit; SQK-LSK109). Sequencing was conducted using an R9.4.1 flow cell on a PromethION P48 instrument, and base calling was performed in real time using MinKNOW Core v3.6.8. An average read *N*_50_ value of 17,366 bp was obtained. The Illumina reads were trimmed using Trimmomatic v0.39 (5), and the final quality was manually checked using FastQ Screen v0.14 (6). The Nanopore reads were also filtered and trimmed using NanoLyse v1.2 and NanoFilt v2.6 (7), with a threshold quality score of 8. The sequencing depths for strains 18, 7, and 20 were, respectively, 17×, 20×, and 21× for Illumina and 86×, 24×, and 36× for Nanopore. The Illumina and Nanopore reads were then used as the input for a hybrid *de novo* genome assembly and circularization of the replicons using the Unicycler v04.9 pipeline (8) with default parameters. Manual finishing was performed based on similarity searches and synteny block detection using the Nanopore assembly sequence of each strain. Each of the 3 fully assembled genomes contains a single circular chromosome and plasmids (Table 1). They comprise, respectively, 5,746,647, 5,686,305, and 5,811,799 bases, with G+C contents of 35.3%, 35.5%, and 35.4%. Genome annotation was performed using the NCBI Prokaryotic Genome Annotation pipeline v5.1 (9). The similarity was assessed by genomic comparison with the sequences (DNA–DNA hybridization) of type strains using the Genome-to Genome Distance Calculator (GGDC) Web server (http://ggdc.dsmz.de/). This work reveals the misidentification of B. cereus group species by MALDI-TOF and shows that several species are responsible for bacteremia in preterm infants.

**TABLE 1:** Genomic characteristics and accession numbers of the bacterial isolates Bacillus cereus strain 18, Bacillus luti strain 7, and Bacillus paranthracis strain 20^*a*^

Characteristic	Data for strain:		
	18	7	20
Bacterial species^*b*^	Bacillus cereus	Bacillus luti	Bacillus paranthracis
MLST	ST74	ST24	ST26
Isolation date (yr/mo/day)	2013/08/11	2017/03/31	2016/07/04
Country: city	France: Nice	France: Paris	France: Marseille
BioSample accession no.	SAMEA8000806	SAMEA6854266	SAMEA6854265
GenBank assembly accession no.	GCA_905221555	GCA_905221595	GCA_903545225
GenBank accession no.	CAJNDP000000000	CAJNDR000000000	CAGVOV000000000
Total no. of reads (ONT)	118,533	258,028	252,077
Total no. of reads (Illumina)	9,041,928	8,831,560	8,955,974
Genome size (bp)	5,746,647	5,686,305	5,811,799
Topology	Circular	Circular	Circular
No. of full genome sequences (chromosome + plasmids)	1 + 6	1 + 3	1 + 4
*N*_50_ (bp)	5,318,884	5,469,117	5,275,862
G+C content (%)	35.3	35.5	35.4
Total no. of genes	5,911	5,761	5,963
No. of protein-coding genes	5,640	5,488	5,543
No. of rRNAs	42	42	42
No. of 5S, 23S, and 16S rRNAs	14	14	14

a MLST, multilocus sequence type; ST, sequence type; ONT, Oxford Nanopore Technologies.

b Strain identification by ≥96% similarity or ≥70% with digital DNA-DNA hybridization (dDDH; dDDH values of >70% are considered an indication that the tested organism belongs to a different species than the type strain[s] used as the reference[s]).

### Data availability.

The three genome sequences and related metadata have been deposited in the GenBank database under accession numbers CAJNDP000000000, CAJNDR000000000, and CAGVOV000000000 for strains 18, 7, and 20, respectively. The raw reads were deposited in the SRA database under accession numbers SRR15372309, SRR15372310, and SRR15372308 (Illumina) and SRR15372306, SRR15372307, and SRR15372305 (Oxford Nanopore Technologies; ONT), respectively, for strains 18, 7, and 20.
